# From self-efficacy to engineering thinking: the mediating role of student engagement among undergraduate engineering students in China

**DOI:** 10.3389/fpsyg.2026.1761444

**Published:** 2026-03-20

**Authors:** Fang Chen, Gede Rasben Dantes, Kadek Rihendra Dantes, Dessy Seri Wahyuni

**Affiliations:** 1 Qingdao Hengxing University of Science and Technology, Qingdao, China; 2 Doctoral Program in Education, Universitas Pendidikan Ganesha, Singaraja, Indonesia; 3 Information System Department, Universitas Pendidikan Ganesha, Singaraja, Indonesia; 4 Mechanical Engineering Education Department, Universitas Pendidikan Ganesha, Singaraja, Indonesia; 5 Informatics Education Department, Universitas Pendidikan Ganesha, Singaraja, Indonesia

**Keywords:** DBL-implemented course context, educational psychology, engineering thinking, engineering undergraduates, self-efficacy, student engagement

## Abstract

Engineering thinking has become a central goal of undergraduate engineering education, yet the psychological pathways through which it emerges in design-oriented course contexts are not well understood. This study examined how self-efficacy is associated with engineering thinking among engineering undergraduates in China and tested student engagement as a mediator of this link in courses incorporating design-based learning (DBL) activities. A cross-sectional questionnaire survey was completed by 407 students enrolled in 12 engineering-related programs at an applied university in eastern China during the second semester of the 2024–2025 academic year. The questionnaire assessed academic self-efficacy, behavioral, emotional, and cognitive engagement in course design activities, and engineering thinking. Data were analyzed using descriptive statistics, bivariate correlations, and partial least squares structural equation modeling (PLS-SEM) in SmartPLS 4.1.1.2 with 5,000 bootstrap resamples. The measurement model satisfied widely accepted criteria for indicator reliability, internal consistency, and convergent and discriminant validity. In the structural model, self-efficacy predicted higher levels of student engagement and engineering thinking, and engagement was positively related to engineering thinking. Indirect-effect estimates showed that student engagement transmitted part of the effect of self-efficacy on engineering thinking, indicating partial mediation. These findings suggest that students who feel more efficacious are more likely to invest behavioral, emotional, and cognitive resources in design-oriented learning tasks, and that this deeper involvement is linked to more advanced engineering thinking. The study highlights engagement as a key psychological mechanism connecting students’ self-beliefs with complex engineering outcomes in DBL-implemented course settings.

## 1 Introduction

In contemporary engineering education, expectations have shifted from training students to follow prescribed procedures toward preparing graduates to address complex, ill-structured problems. Within this shift, engineering thinking—often framed as an integration of analytical, creative, systematic, and reflective reasoning—has become a prominent target of curriculum reform (Dym et al., 2005; Prince and Felder, 2006). Design-based learning (DBL) and related project-oriented pedagogies have been adopted to support this agenda by engaging students in iterative cycles of problem framing, ideation, prototyping, testing, and refinement around authentic tasks (English and Kitsantas, 2013; Gómez Puente et al., 2015; Wei et al., 2023). Evidence from educational psychology also indicates that the outcomes of active, design-focused environments are closely tied to students’ motivational resources and their behavioral, emotional, and cognitive involvement in learning activities (Fredricks et al., 2004, 2016; Lam et al., 2012; Schaufeli et al., 2002). In parallel, self-efficacy, or students’ beliefs in their capability to organize and carry out the actions needed for success, has emerged as a central predictor of engagement and achievement in engineering and higher education (Bandura, 1997; Chemers et al., 2001; Eccles and Wigfield, 2002; Mamaril et al., 2016; Wang and Zhang, 2024).

A substantial body of work in higher education shows that students who report stronger academic self-efficacy also report higher engagement, and review evidence suggests that engagement can help explain part of the association between self-efficacy and academic outcomes such as achievement or adjustment (Adams et al., 2020; Honicke and Broadbent, 2016; Luo et al., 2023). At the same time, much of this literature relies on broad outcomes (e.g., grades or course satisfaction) rather than engineering thinking as a domain-specific higher-order capability, and engagement is often examined as an outcome of instructional innovation rather than as a proximal pathway linking self-beliefs to complex engineering outcomes. Evidence from Chinese undergraduate engineering programs remains comparatively limited, particularly in applied universities where large enrollments and intensive assessment routines may shape both engagement and efficacy beliefs (Luo et al., 2023; Wang and Zhang, 2024). To address this gap, the present study tests a mediation model in which academic self-efficacy is linked to engineering thinking both directly and indirectly through multidimensional student engagement among engineering undergraduates enrolled in DBL-implemented courses at a teaching-focused university in eastern China. In this study, DBL is treated as the instructional context that defines the sampling frame, while the focal relationships concern the psychological pathway from self-efficacy to engineering thinking via engagement.

## 2 Literature review

### 2.1 Engineering thinking in design-oriented engineering courses

Engineering thinking is increasingly described as a higher-order capacity that integrates analytical, creative, systematic, and reflective reasoning to address complex, ill-structured problems (Dym et al., 2005; Prince and Felder, 2006). Recent work in STEM and engineering education has operationalized engineering thinking through dimensions such as systems thinking, critical thinking, and creative problem solving, and has shown that these skills can be strengthened when engineering design tasks are embedded in science or engineering curricula (English and Kitsantas, 2013; Wei et al., 2023). Quasi-experimental and design-based studies suggest that design-oriented engineering learning can produce better learning outcomes than traditional lecture-based instruction and that iterative design projects provide a particularly fertile context for developing engineering thinking, especially when they require students to integrate conceptual understanding with constraints and trade-offs typical of engineering practice (Gómez Puente et al., 2015; Hmelo-Silver, 2004). However, much of this work emphasizes instructional formats and outcome gains, while the psychological processes through which students convert design experiences into stronger engineering thinking remain under-specified. Beyond its overlap with general critical thinking and problem-solving, engineering thinking is situated in design and systems contexts in which learners must coordinate constraints, trade-offs, and stakeholder considerations. In contemporary design contexts, this competence also involves critically evaluating proposed solutions by checking assumptions, constraints, and evidence before adopting them. In survey-based studies, self-report measures are commonly used to capture students’ perceived competence in these forms of reasoning, which should be interpreted as perceived capability rather than direct performance.

### 2.2 Academic self-efficacy and student engagement

Academic self-efficacy, grounded in Social Cognitive Theory, refers to students’ beliefs in their capability to organize and execute the actions required to attain desired academic outcomes (Bandura, 1997). In engineering education, domain-specific engineering self-efficacy predicts grades, persistence intentions, and performance on engineering tasks, even when prior achievement is controlled (Chemers et al., 2001; Eccles and Wigfield, 2002; Mamaril et al., 2016). Students who hold stronger self-efficacy beliefs are more inclined to set ambitious goals, invest sustained effort, and persist when they encounter difficulties—patterns that are especially salient in demanding design-based settings, where tasks are complex and outcomes cannot be fully anticipated (English and Kitsantas, 2013; Linnenbrink and Pintrich, 2003).

Student engagement is widely described as a multidimensional construct with behavioral, emotional, and cognitive facets (Fredricks et al., 2004), and higher levels of engagement have been consistently linked to better academic performance and adjustment in both school and university contexts (Fredricks et al., 2016; Lam et al., 2012; Schaufeli et al., 2002). A growing body of empirical work in higher education shows that self-efficacy is positively related to behavioral, emotional, and cognitive engagement. Studies in traditional and blended learning environments report that students with stronger academic self-efficacy are more likely to participate actively, persist in their studies, and use strategic approaches to learning (Azila-Gbettor et al., 2021). Research conducted in Asian contexts similarly indicates that self-efficacy supports students’ willingness to engage deeply with demanding academic tasks, including those in professional and applied programs (Luo et al., 2023; Richardson et al., 2012; Wang and Zhang, 2024). Overall, empirical findings and review evidence converge on the view that self-efficacy functions as a motivational resource that is consistently associated with students’ behavioral participation, affective involvement, and strategic learning in higher education settings (Linnenbrink and Pintrich, 2003; Richardson et al., 2012).

### 2.3 Self-efficacy, engagement, and engineering thinking

At the intersection of these lines of work, recent studies have begun to examine engagement as a process variable in models of design-oriented learning. Wei et al. (2023), for example, found that cognitive engagement mediated the relationship between design-based engineering learning and engineering students’ learning outcomes, suggesting that engagement can serve as a conduit through which design-rich pedagogies relate to learning outcomes. Research on problem-based and project-based learning similarly indicates that students who are more deeply engaged, particularly at the cognitive level, tend to produce higher-quality solutions and demonstrate more sophisticated forms of thinking in engineering and STEM domains (English and Kitsantas, 2013; Fredricks et al., 2004; Prince and Felder, 2006).

Beyond DBL specifically, broader higher education studies have modeled engagement as a mediator between self-efficacy and academic outcomes. Findings from diverse disciplines and contexts show that learning engagement partially explains how self-efficacy relates to grades, perceived learning, and academic satisfaction (Adams et al., 2020; Luo et al., 2023). These studies align with the view that self-efficacy shapes internal processes—such as effort regulation, strategy use, and emotional responses—which then manifest as engagement patterns and, ultimately, as performance (Benlahcene et al., 2024; Linnenbrink and Pintrich, 2003). However, many existing models focus on general academic outcomes or treat engagement primarily as an endpoint, leaving less clarity on how engagement operates as a proximal process linking self-beliefs with domain-specific higher-order outcomes such as engineering thinking. This study extends prior higher education evidence by examining the self-efficacy–engagement pathway with engineering thinking specified as the distal outcome within DBL-implemented undergraduate engineering course contexts in China.

### 2.4 Theoretical framework and hypotheses

Social Cognitive Theory (SCT) posits that human functioning reflects reciprocal interactions among personal factors, behavior, and environmental conditions (Bandura, 1997). Within SCT, academic self-efficacy reflects students’ judgments about their capability to organize and execute actions needed to attain academic goals; in demanding engineering learning tasks, stronger efficacy beliefs are typically associated with greater effort investment, persistence, and adaptive coping (Chemers et al., 2001; Eccles and Wigfield, 2002; Mamaril et al., 2016). In the present study, DBL is treated as the course context in which participants experienced iterative design activities, while the focal theoretical claim concerns how self-beliefs relate to engagement processes and higher-order outcomes within that context.

Multidimensional engagement models conceptualize engagement as behavioral participation, emotional involvement, and cognitive strategy use, and position engagement as a proximal process through which motivational beliefs are expressed in day-to-day learning activity (Fredricks et al., 2004, 2016; Linnenbrink and Pintrich, 2003). Some scholars have proposed agentic engagement as an additional facet that reflects students’ proactive contribution to instructional activities (Reeve and Tseng, 2011). Consistent with SCT and engagement frameworks, students with higher academic self-efficacy are expected to report higher engagement in DBL-implemented course activities, and students who report higher engagement are expected to report stronger engineering thinking.

On this basis, academic self-efficacy is expected to be positively associated with engineering thinking both directly and indirectly through student engagement. Higher self-efficacy may be reflected in greater willingness to persist in open-ended design tasks and to try alternative solution pathways, while engagement captures the more immediate behavioral, emotional, and cognitive investment through which design experiences are connected to perceived higher-order capability (English and Kitsantas, 2013; Fredricks et al., 2004).

Based on this reasoning and the empirical evidence reviewed above, the study tests the following hypotheses:

*H1*: Academic self-efficacy positively predicts student engagement in DBL-implemented undergraduate engineering courses among engineering undergraduates in China.

*H2*: Student engagement positively predicts engineering thinking among undergraduate engineering students.

*H3*: Academic self-efficacy positively predicts engineering thinking among undergraduate engineering students.

*H4*: Student engagement mediates the relationship between academic self-efficacy and engineering thinking, such that self-efficacy exerts an indirect effect on engineering thinking through engagement.

**Figure 1:**
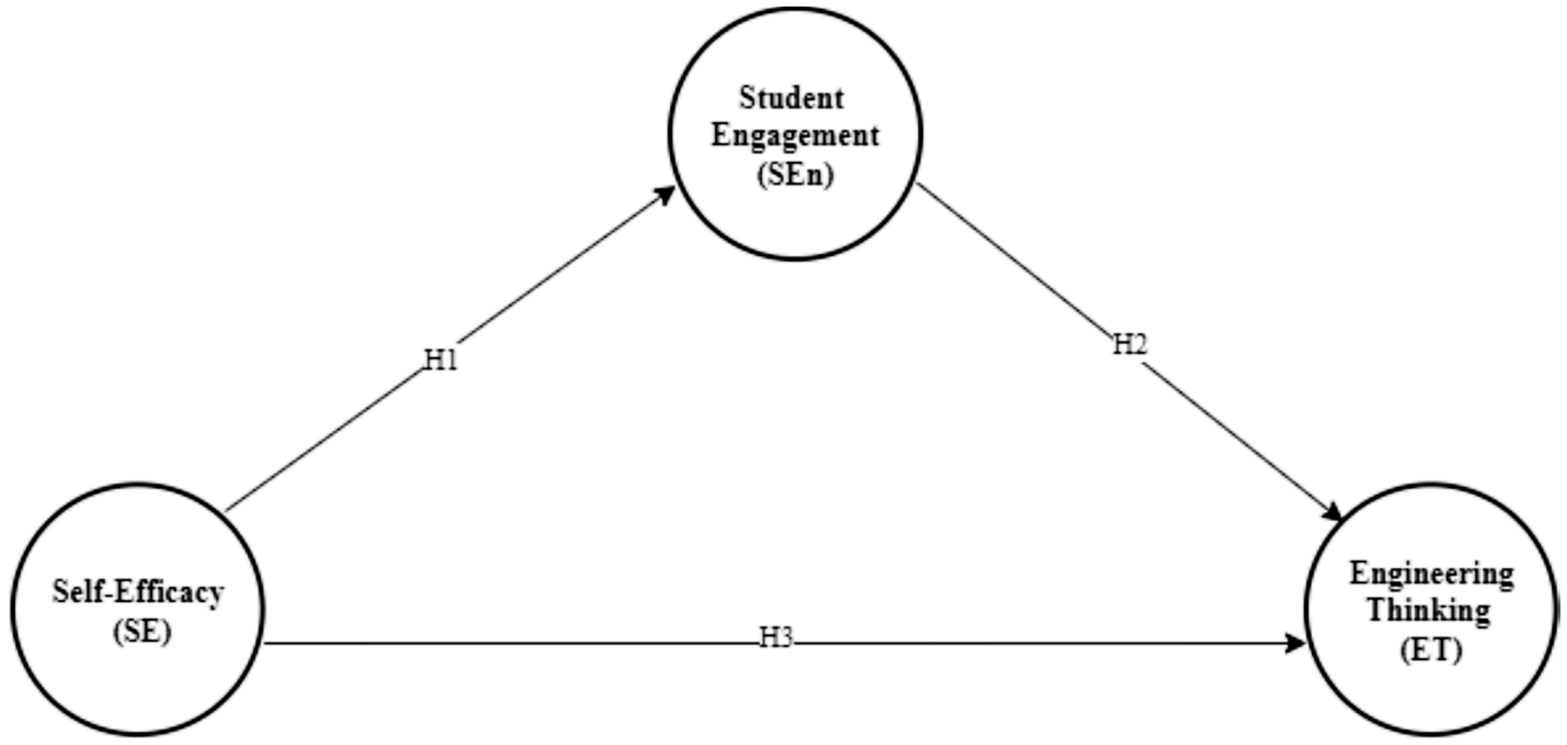
Hypothesized mediation model. Academic self-efficacy (SE) is specified as a predictor of student engagement (SEn) and engineering thinking (ET), and SEn is specified as a mediator linking SE to ET (H1–H4). Design-based learning (DBL) is treated as the instructional context defining the sampling frame (DBL-implemented courses) rather than an antecedent in the structural paths.

The hypothesized mediation model is summarized in Figure 1, which illustrates the proposed direct paths from self-efficacy to student engagement and engineering thinking, as well as the indirect pathway from self-efficacy to engineering thinking via engagement.

These hypotheses are examined using a higher-order PLS-SEM mediation model in which self-efficacy, student engagement, and engineering thinking are each modeled as reflective higher-order constructs with multiple first-order dimensions.

## 3 Method

### 3.1 Participants

Participants were undergraduate students enrolled in 12 engineering-related programs at Qingdao Hengxing University of Science and Technology (hereafter, Hengxing University)—Electrical Engineering and Automation, Mechanical Engineering, Vehicle Engineering, Automotive Service Engineering, Robotics Engineering, Intelligent Manufacturing Engineering, Software Engineering, Network Engineering, Artificial Intelligence, Engineering Cost, Civil Engineering, and Printing Engineering (Table 1). Using class lists provided by program coordinators, intact classes that had completed at least one course design project involving problem exploration, design generation, prototype iteration, and reflection/evaluation during the current or previous semester were invited to participate. After data-quality screening, 407 valid responses were retained from 420 submissions. The sample included students from Years 1–4; demographic distributions are reported in Table 1.

**Table 1:** Sample characteristics (gender, year, major).

Variable	Category	*n*	%
Gender	Male	244	59.95
	Female	163	40.05
	Total	407	100
Year level	Freshman (1st year)	95	23.34
	Sophomore (2nd year)	145	35.63
	Junior (3rd year)	115	28.26
	Senior (4th year)	52	12.78
	Total	407	100
Major	Electrical engineering and automation	44	10.81
	Mechanical engineering	42	10.32
	Vehicle engineering	33	8.11
	Automotive service engineering	27	6.63
	Robotics engineering	31	7.62
	Intelligent manufacturing engineering	33	8.11
	Software engineering	56	13.76
	Network engineering	38	9.34
	Artificial intelligence	36	8.85
	Engineering cost	25	6.14
	Civil engineering	25	6.14
	Printing engineering	17	4.18
	Total	407	100

Percentages may not sum to 100 due to rounding.

### 3.2 Measurement instruments

Self-efficacy, student engagement, and engineering thinking were assessed using 36 items. The finalized item set was developed through an iterative adaptation process informed by established measures and prior studies. The questionnaire was prepared in bilingual form. Items were initially drafted in English, translated into Chinese by bilingual experts, and then back-translated by an independent bilingual reviewer to ensure conceptual equivalence. Any discrepancies were resolved through expert discussion. All responses were recorded on a five-point Likert scale ranging from 1 (strongly disagree) to 5 (strongly agree). A pilot test with engineering students was conducted to examine item clarity and preliminary internal consistency, and the results supported use of the instrument in the main survey. The full wording of all items, together with their dimension labels and source/adaptation notes, is provided in Supplementary Table S1.

#### 3.2.1 Self-efficacy (SE)

In this study, academic self-efficacy was modeled as a higher-order construct with five first-order dimensions—task-specific, context-specific, affective, time management, and self-regulation efficacy. Each dimension was measured with three items, yielding 15 items in total. Sample items reflected students’ confidence in completing learning tasks, adapting to varying instructional conditions, and sustaining focus and regulation in demanding engineering-learning contexts. The scale was informed by prior work on academic and engineering self-efficacy, which has reported good internal consistency in higher education samples (Cronbach’s alpha typically above 0.80). Higher scores indicate stronger self-efficacy beliefs. Item wording was informed by prior research on academic and engineering self-efficacy in higher education (e.g., Bandura, 1997; Chemers et al., 2001; Mamaril et al., 2016).

#### 3.2.2 Student engagement (SEn)

Student engagement was modeled as a higher-order construct with three first-order dimensions—behavioral, emotional, and cognitive engagement—based on the multidimensional framework proposed by Fredricks et al. (2004). Each dimension was measured with three items (nine items in total). Behavioral engagement items captured effort and participation in course design activities (e.g., putting considerable effort into the design tasks in the course). Emotional engagement items reflected students’ interest and enjoyment of the learning activities. Cognitive engagement items reflected deep understanding, self-generated explanation, and effortful processing of learning content, including attempts to connect new learning with prior engineering knowledge. Higher scores indicate higher levels of engagement in DBL-implemented courses. The three-facet specification and item wording were informed by multidimensional engagement frameworks and higher education engagement research (e.g., Fredricks et al., 2004, 2016; Reeve and Tseng, 2011).

#### 3.2.3 Engineering thinking (ET)

Engineering thinking was conceptualized as a higher-order construct with four first-order dimensions—analytical, creative, systematic, and reflective thinking—drawing on operationalization’s in engineering and STEM education. Each dimension was measured with three items (12 items in total). Illustrative items reflected identifying core technical issues, generating multiple possible approaches to engineering problems, considering relationships among components and systems, and reflecting on process and decisions after completing engineering tasks. Content validity was supported through expert review, and the pilot administration indicated acceptable preliminary reliability. Higher scores indicate stronger self-reported engineering thinking. The four-dimension operationalization and item wording were informed by engineering design thinking scholarship and related STEM education work (e.g., Dym et al., 2005; Prince and Felder, 2006; Wei et al., 2023).

### 3.3 Procedure and ethics

Data collection was conducted during the second semester of the 2024–2025 academic year using a structured online questionnaire administered via Wenjuanxing (Questionnaire Star). The survey was completed during regular class sessions in DBL-implemented engineering courses. Course instructors shared the survey link/QR code, and students completed the questionnaire on their own devices. Participation was voluntary and unrelated to course grades. Participants could stop at any point by exiting the survey without submitting a response. Before starting the questionnaire, participants read an information sheet and provided electronic informed consent; only consenting students could proceed. No personally identifiable information was collected.

Eligibility was verified using a single yes/no item confirming prior exposure to a course design project that included all four DBL phases (problem exploration, design generation, prototype iteration, and reflection/evaluation). This item was used only to define eligibility and was not intended to assess DBL implementation quality or fidelity. Specifically, the screening item asked whether the student had completed at least one course design project during the current or previous semester that included all four phases—problem exploration, design generation, prototype iteration, and reflection/evaluation (Yes/No); only “Yes” responses were retained. Recruitment focused on intact classes that had completed at least one such project during the current or previous semester to minimize context misclassification. The screening item served as an inclusion criterion rather than a measure of DBL implementation quality; therefore, variation in implementation fidelity across courses and instructors was not analyzed. Most students completed the questionnaire in approximately 10 min. The platform required complete responses prior to submission; thus, no system-generated missing data were recorded. Responses indicative of careless responding (e.g., straight-lining across consecutive item blocks) or implausibly short completion times (<3 min) were removed. In total, 420 submissions were received and 407 valid cases were retained for analysis. Thus, the findings pertain to students with verified DBL exposure and do not estimate the effects of DBL intensity or fidelity.

The study protocol was reviewed by the Research Ethics Committee of Universitas Pendidikan Ganesha. The study was conducted in accordance with the local legislation and institutional requirements, and the ethics review was handled in accordance with institutional procedures.

### 3.4 Data analysis

Data were screened in IBM SPSS Statistics 27.0 to verify data quality (e.g., response-pattern flags and completion time), identify outliers, and examine basic distributional characteristics. Descriptive statistics and Pearson correlations were computed for the three higher-order constructs. Partial least squares structural equation modeling (PLS-SEM) was conducted in SmartPLS 4.1.1.2 to evaluate the measurement and structural models. Self-efficacy, student engagement, and engineering thinking were specified as reflective higher-order constructs with reflective first-order dimensions and estimated using a disjoint two-stage approach. In Stage 1, the PLS algorithm was used to estimate the first-order reflective measurement models and obtain latent variable scores for each first-order dimension. In Stage 2, these scores were used as indicators of the higher-order constructs, and the higher-order measurement model and the structural model were estimated using PLSc. Statistical inference for direct and indirect effects was based on bootstrapping with 5,000 subsamples using bias-corrected and accelerated (BCa) confidence intervals (two-tailed, *α* = 0.05) with a fixed random seed. Following Kock’s (2015) full collinearity approach, we exported construct-level latent variable scores and estimated three linear regressions (OLS) in SPSS: SE ~ SEn + ET, SEn ~ SE + ET, and ET ~ SE + SEn. This procedure yielded full collinearity VIFs (predictor VIFs) for common method bias assessment, which were evaluated against the 3.3 cutoff.

The measurement models were assessed in terms of indicator reliability, internal consistency reliability (Cronbach’s alpha, rho_A, composite reliability), convergent validity (average variance extracted), collinearity (VIF), and discriminant validity (Fornell–Larcker criterion and heterotrait–monotrait ratios). The structural model was evaluated using path coefficients, coefficients of determination (*R*^2^), and effect sizes (*f*^2^). Out-of-sample predictive performance was examined with the PLSpredict procedure (10-fold cross-validation with 10 repetitions), which provides cross-validated *Q*^2^_predict_ values for the endogenous constructs. These values refer to *Q*^2^_predict_ from PLSpredict (cross-validated predictive relevance) rather than Stone–Geisser’s *Q*^2^ obtained via blindfolding. The hypothesized mediation model is shown in Figure 1.

## 4 Results

### 4.1 Preliminary analyses

Table 2 reports descriptive statistics and Pearson correlations for the three higher-order constructs. On the five-point scale, mean scores for self-efficacy (M = 2.994, SD = 1.076), student engagement (M = 2.992, SD = 1.152), and engineering thinking (M = 2.993, SD = 1.112) were close to the midpoint, indicating moderate perceived levels. Skewness ranged from 0.02 to 0.09 and kurtosis from −1.097 to −1.045, indicating approximately symmetric distributions at the construct level.

**Table 2:** Descriptive statistics and Pearson correlations (*N* = 407).

Variable	M	SD	Skewness	Kurtosis	1	2	3
1. Self-efficacy (SE_mean)	2.994	1.076	0.018	−1.097	1		
2. Student engagement (SEn_mean)	2.992	1.152	0.051	−1.087	0.527**	1	
3. Engineering thinking (ET_mean)	2.993	1.112	0.088	−1.045	0.531**	0.679**	1

Construct means were computed as composite means across indicators; SD reflects between-student variability on the same 5-point scale. Pearson correlations were computed using composite scores, whereas discriminant validity assessments used latent variable correlations from the PLS model.

Correlations among the three constructs were positive and statistically significant (*p* < 0.001). Self-efficacy was moderately correlated with student engagement (*r* = 0.527) and engineering thinking (*r* = 0.531), and student engagement showed a relatively stronger association with engineering thinking (*r* = 0.679). These patterns are consistent with the hypothesized model in which higher self-efficacy is expected to foster greater engagement in learning activities, which in turn is associated with stronger engineering thinking. Full collinearity VIFs ranged from 1.392 to 1.860 (all <3.3) (Table 3, Panel B), providing no indication of substantial common method bias.

**Table 3:** Collinearity diagnostics (VIF) for the higher-order model and full collinearity assessment.

Panel A. Collinearity statistics (VIF) for first-order dimensions used as indicators of the higher-order constructs (Stage 2)	
First-order dimension	VIF
Task-specific self-efficacy (SE_TS)	2.933
Context-specific self-efficacy (SE_CS)	2.863
Affective self-efficacy (SE_AF)	3.002
Time management self-efficacy (SE_TM)	2.723
Self-regulation self-efficacy (SE_SR)	2.798
Behavioral engagement (SEn_BE)	2.952
Emotional engagement (SEn_EM)	2.772
Cognitive engagement (SEn_CO)	2.398
Analytical thinking (ET_AN)	2.527
Creative thinking (ET_CR)	2.628
Systematic thinking (ET_SY)	3.132
Reflective thinking (ET_RE)	2.896

Panel B. Full collinearity VIFs based on construct-level latent variable scores (SPSS regressions)			
Dependent LV score	Predictors	VIF (predictor 1)	VIF (predictor 2)
LVscoresSE	LVscoresSEn, LVscoresET	1.860	1.860
LVscoresSEn	LVscoresSE, LVscoresET	1.395	1.395
LVscoresET	LVscoresSE, LVscoresSEn	1.392	1.392

### 4.2 Measurement model

**Figure 2:**
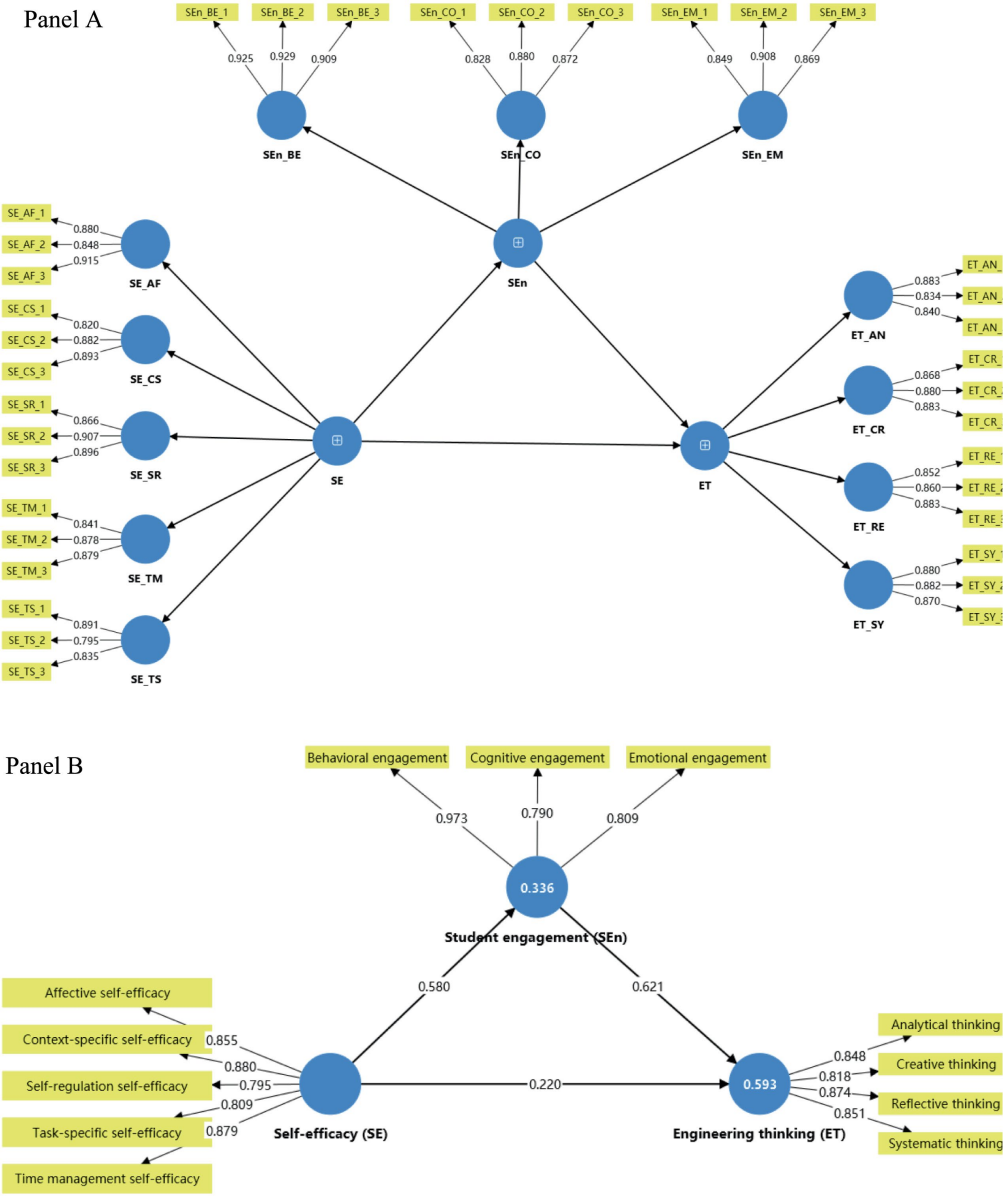
Disjoint two-stage higher-order PLS-SEM model. **(A)** Stage 1 reflective measurement model with indicators and first-order dimensions (standardized outer loadings shown). **(B)** Stage 2 higher-order model in which first-order dimensions serve as indicators of the higher-order constructs, together with the structural paths among SE, SEn, and ET. Standardized path coefficients (*β*) and coefficients of determination (*R*^2^) for endogenous constructs are displayed. Estimates are based on PLSc with bootstrapping (5,000 resamples; bias-corrected and accelerated (BCa) confidence intervals; two-tailed, *α* = 0.05; *N* = 407).

The two-stage higher-order measurement model is depicted in Figure 2 (Panel A, B). The reflective measurement models were first evaluated at the level of the first-order dimensions. As shown in Table 4, all items loaded strongly on their intended dimensions, with standardized loadings ranging from 0.795 to 0.929. All indicators loaded highest on their intended dimensions and met indicator reliability criteria; therefore, all indicators were retained.

**Table 4:** Indicator loadings for first-order dimensions (Stage 1 model).

First-order construct (dimension)	Indicator	Loading
Task-specific self-efficacy (SE_TS)	SE_TS_1	0.891
Task-specific self-efficacy (SE_TS)	SE_TS_2	0.795
Task-specific self-efficacy (SE_TS)	SE_TS_3	0.835
Context-specific self-efficacy (SE_CS)	SE_CS_1	0.820
Context-specific self-efficacy (SE_CS)	SE_CS_2	0.882
Context-specific self-efficacy (SE_CS)	SE_CS_3	0.893
Affective self-efficacy (SE_AF)	SE_AF_1	0.880
Affective self-efficacy (SE_AF)	SE_AF_2	0.848
Affective self-efficacy (SE_AF)	SE_AF_3	0.915
Time management self-efficacy (SE_TM)	SE_TM_1	0.841
Time management self-efficacy (SE_TM)	SE_TM_2	0.878
Time management self-efficacy (SE_TM)	SE_TM_3	0.879
Self-regulation self-efficacy (SE_SR)	SE_SR_1	0.866
Self-regulation self-efficacy (SE_SR)	SE_SR_2	0.907
Self-regulation self-efficacy (SE_SR)	SE_SR_3	0.896
Behavioral engagement (SEn_BE)	SEn_BE_1	0.925
Behavioral engagement (SEn_BE)	SEn_BE_2	0.929
Behavioral engagement (SEn_BE)	SEn_BE_3	0.909
Emotional engagement (SEn_EM)	SEn_EM_1	0.849
Emotional engagement (SEn_EM)	SEn_EM_2	0.908
Emotional engagement (SEn_EM)	SEn_EM_3	0.869
Cognitive engagement (SEn_CO)	SEn_CO_1	0.828
Cognitive engagement (SEn_CO)	SEn_CO_2	0.880
Cognitive engagement (SEn_CO)	SEn_CO_3	0.872
Analytical thinking (ET_AN)	ET_AN_1	0.883
Analytical thinking (ET_AN)	ET_AN_2	0.834
Analytical thinking (ET_AN)	ET_AN_3	0.840
Creative thinking (ET_CR)	ET_CR_1	0.868
Creative thinking (ET_CR)	ET_CR_2	0.880
Creative thinking (ET_CR)	ET_CR_3	0.883
Systematic thinking (ET_SY)	ET_SY_1	0.880
Systematic thinking (ET_SY)	ET_SY_2	0.882
Systematic thinking (ET_SY)	ET_SY_3	0.870
Reflective thinking (ET_RE)	ET_RE_1	0.852
Reflective thinking (ET_RE)	ET_RE_2	0.860
Reflective thinking (ET_RE)	ET_RE_3	0.883

Internal consistency and convergent validity for the first-order dimensions were satisfactory. As shown in Table 5, Cronbach’s alpha values ranged from 0.793 to 0.911; rho_A ranged from 0.803 to 0.913; composite reliability ranged from 0.879 to 0.944; average variance extracted (AVE) values ranged from 0.707 to 0.848, indicating that each dimension explained more than half of the variance in its indicators.

**Table 5:** Internal consistency and convergent validity for first-order dimensions (Stage 1 model).

First-order construct (dimension)	Cronbach’s *α*	rho_A	CR	AVE
Task-specific self-efficacy (SE_TS)	0.793	0.803	0.879	0.707
Context-specific self-efficacy (SE_CS)	0.833	0.839	0.900	0.749
Affective self-efficacy (SE_AF)	0.856	0.860	0.913	0.777
Time management self-efficacy (SE_TM)	0.834	0.836	0.900	0.751
Self-regulation self-efficacy (SE_SR)	0.869	0.871	0.920	0.792
Behavioral engagement (SEn_BE)	0.911	0.913	0.944	0.848
Emotional engagement (SEn_EM)	0.848	0.853	0.908	0.767
Cognitive engagement (SEn_CO)	0.825	0.829	0.895	0.740
Analytical thinking (ET_AN)	0.812	0.814	0.889	0.727
Creative thinking (ET_CR)	0.850	0.851	0.909	0.769
Systematic thinking (ET_SY)	0.850	0.851	0.909	0.770
Reflective thinking (ET_RE)	0.832	0.834	0.899	0.749

Collinearity diagnostics showed no serious multicollinearity problems. The VIF values for the first-order dimensions used as indicators of the higher-order constructs ranged from 2.40 to 3.13 (rounded), all below 3.3 (Table 5, Panel A).

Reliability, convergent validity, and discriminant validity at the higher-order construct level are summarized in Table 6 (Panels A–C). The higher-order loadings of the first-order dimensions on their respective constructs are reported in Table 6 (Panel D). The three higher-order constructs—self-efficacy (SE), student engagement (SEn), and engineering thinking (ET)—were then estimated in the second-stage model using the latent scores of their first-order dimensions as indicators. The standardized loadings of the first-order dimensions on their respective higher-order constructs were all high, ranging from 0.790 to 0.973. Cronbach’s alpha values for ET, SE, and SEn were 0.911, 0.926, and 0.893, respectively; rho_A values ranged from 0.906 to 0.927, and composite reliability values from 0.895 to 0.925. AVE values were 0.720 for ET, 0.713 for SE, and 0.742 for SEn, again exceeding the 0.50 criterion and supporting convergent validity at the construct level.

**Table 6:** Reliability and discriminant validity for higher-order constructs (Stage 2 model).

Panel A. Internal consistency and convergent validity				
Construct	Cronbach’s *α*	*ρ*A	CR (*ρ*C)	AVE
Self-efficacy (SE)	0.926	0.927	0.925	0.713
Student engagement (SEn)	0.893	0.906	0.895	0.742
Engineering thinking (ET)	0.911	0.912	0.911	0.720

Panel B. Fornell–Larcker criterion (square roots of AVE on the diagonal)			
Construct	Self-efficacy (SE)	Student engagement (SEn)	Engineering thinking (ET)
Self-efficacy (SE)	0.844		
Student engagement (SEn)	0.580	0.861	
Engineering thinking (ET)	0.580	0.749	0.848

Panel C. HTMT ratios between higher-order constructs			
Construct	Self-efficacy (SE)	Student engagement (SEn)	Engineering thinking (ET)
Self-efficacy (SE)			
Student engagement (SEn)	0.577		
Engineering thinking (ET)	0.579	0.750	

Panel D. Higher-order loadings of first-order dimensions (Stage 2 model)					
Higher-order construct	Dimension (code)	Loading	STDEV	*t*	*p*
Self-efficacy (SE)	Task-specific self-efficacy (SE_TS)	0.809	0.038	21.383	<0.001
	Context-specific self-efficacy (SE_CS)	0.880	0.037	23.587	<0.001
	Affective self-efficacy (SE_AF)	0.855	0.034	25.181	<0.001
	Time management self-efficacy (SE_TM)	0.879	0.034	25.939	<0.001
	Self-regulation self-efficacy (SE_SR)	0.795	0.038	20.766	<0.001
Student engagement (SEn)	Behavioral engagement (SEn_BE)	0.973	0.020	48.135	<0.001
	Emotional engagement (SEn_EM)	0.809	0.026	30.625	<0.001
	Cognitive engagement (SEn_CO)	0.790	0.030	26.397	<0.001
Engineering thinking (ET)	Analytical thinking (ET_AN)	0.848	0.027	31.667	<0.001
	Creative thinking (ET_CR)	0.818	0.028	29.583	<0.001
	Systematic thinking (ET_SY)	0.851	0.026	33.178	<0.001
	Reflective thinking (ET_RE)	0.874	0.024	36.533	<0.001

Entries are latent variable correlations from the Stage 2 PLSc model. The correlations are extremely close (SE–SEn = 0.58008; SE–ET = 0.58009; SEn–ET = 0.74879) and are shown to three decimals for readability.

Discriminant validity was examined using both the Fornell–Larcker criterion and heterotrait–monotrait ratios (HTMT). For the higher-order constructs, the square roots of the AVE (0.848 for ET, 0.844 for SE, and 0.861 for SEn) were larger than the corresponding inter-construct correlations, satisfying the Fornell–Larcker criterion (Table 6, Panel B). HTMT values between the three constructs ranged from 0.577 to 0.750 (Table 6, Panel C), all below the recommended 0.85 cutoff, which further supports discriminant validity.

Taken together, these results indicate that the first-order dimensions and the three higher-order constructs—self-efficacy, student engagement, and engineering thinking—are measured reliably and represent empirically distinct yet related constructs. On this basis, the structural relations and mediation hypotheses were tested in the structural model. With a single predictor of SEn in the structural model, the standardized coefficient for SE → SEn can be numerically close to the corresponding latent correlation; these statistics are reported for different purposes. The Pearson correlations in Table 2 are based on composite mean scores, whereas the latent correlations and path coefficients are based on the disjoint two-stage latent variable scores estimated in SmartPLS (with PLSc correction), which can lead to non-trivial differences in magnitude.

### 4.3 Structural model and mediation

The structural model was evaluated using PLSc estimates from the second-stage higher-order model. As reported in Table 7, all three structural paths were positive and statistically significant. Self-efficacy (SE) had a moderate effect on student engagement (SEn) (*β* = 0.580, *t* = 15.376, *p* < 0.001), and student engagement in turn had a strong effect on engineering thinking (ET) (*β* = 0.621, *t* = 13.329, *p* < 0.001). Self-efficacy also retained a smaller but still significant direct effect on engineering thinking (*β* = 0.220, *t* = 4.368, *p* < 0.001). Together, SE explained 33.6% of the variance in SEn (*R*^2^ = 0.336), and SE and SEn jointly accounted for just over half of the variance in ET (*R*^2^ = 0.593). Effect size estimates indicated that SE had a large effect on SEn (*f*^2^ = 0.507), SEn had a large effect on ET (*f*^2^ = 0.629), and SE had a small-to-moderate direct effect on ET (*f*^2^ = 0.079) (Table 8).

**Table 7:** Structural model results for the higher-order mediation model (*N* = 407).

Path	*β* (original sample)	STDEV	*t*	*p*	95% BCa CI LL	95% BCa CI UL	*f* ^2^	*R*^2^ (endogenous)
Self-efficacy (SE) → student engagement (SEn)	0.580	0.038	15.376	<0.001	0.503	0.650	0.507	SEn: 0.336
Student engagement (SEn) → engineering thinking (ET)	0.621	0.047	13.329	<0.001	0.527	0.706	0.629	ET: 0.593
Self-efficacy (SE) → engineering thinking (ET)	0.220	0.050	4.368	<0.001	0.121	0.317	0.079	—

**Table 8:** Indirect, direct, and total effects of self-efficacy on engineering thinking via student engagement.

Effect	Path	*β*	STDEV	*t*	*p*	95% BCa CI LL	95% BCa CI UL	VAF
Direct effect	SE → ET	0.220	0.050	4.368	<0.001	0.121	0.317	—
Indirect effect	SE → SEn → ET	0.360	0.036	9.994	<0.001	0.293	0.435	0.621
Total effect	SE → ET (total)	0.580	0.038	15.123	<0.001	0.500	0.650	—

The total effect equals the sum of the direct and indirect effects (0.220 + 0.360 = 0.580). With a single predictor of SEn, the standardized path SE → SEn can be numerically close to the latent correlation; all values are rounded to three decimals.

Bootstrapping results indicated that the indirect effect of self-efficacy on engineering thinking via student engagement was positive and significant (*β* = 0.360, *p* < 0.001), while the direct effect remained positive and significant (*β* = 0.220, *p* < 0.001). The resulting total effect was *β* = 0.580 (*p* < 0.001). Because both direct and indirect effects were significant and in the same direction, the mediation pattern is best characterized as complementary partial mediation (Zhao et al., 2010). The total effect reflected the sum of the direct and indirect effects (reported to three decimals). Because several estimates round to the same value (e.g., 0.580), we report coefficients to three decimals for readability; small differences may emerge when reporting additional decimals.

### 4.4 Predictive assessment (PLSpredict)

Out-of-sample predictive performance was examined using PLSpredict with 10-fold cross-validation and 10 repetitions. At the construct level, the *Q*^2^_predict_ values were positive for both endogenous constructs (*Q*^2^_predict(SEn)_ = 0.280; *Q*^2^_predict(ET)_ = 0.281), indicating predictive relevance for student engagement and engineering thinking (Table 9). At the indicator level (i.e., the first-order dimensions serving as indicators in the second-stage higher-order model), all seven indicators used to predict the two endogenous constructs also showed positive *Q*^2^_predict_ values (range = 0.179–0.315) (Table 10).

**Table 9:** Construct-level *Q*^2^_predict (SEn, ET)_.

Construct	*Q* ^2^_predict_	RMSE	MAE
Student engagement (SEn)	0.280	0.853	0.713
Engineering thinking (ET)	0.281	0.852	0.718

**Table 10:** Indicator-level comparison (PLS vs. LM; RMSE/MAE and Δ).

Indicator	*Q* ^2^_predict_	PLS_RMSE	PLS_MAE	LM_RMSE	LM_MAE	ΔRMSE (PLS-LM)	ΔMAE (PLS-LM)
Behavioral engagement (SEn_BE)	0.315	0.831	0.711	0.833	0.704	−0.002	0.007
Emotional engagement (SEn_EM)	0.179	0.908	0.770	0.915	0.780	−0.007	−0.011
Cognitive engagement (SEn_CO)	0.183	0.907	0.771	0.911	0.775	−0.005	−0.004
Analytical thinking (ET_AN)	0.201	0.896	0.752	0.901	0.759	−0.005	−0.008
Creative thinking (ET_CR)	0.201	0.897	0.758	0.898	0.755	−0.001	0.003
Systematic thinking (ET_SY)	0.235	0.877	0.741	0.880	0.739	−0.003	0.001
Reflective thinking (ET_RE)	0.248	0.870	0.742	0.874	0.741	−0.004	0.001

Prediction error benchmarks further compared PLS-SEM with a linear model (LM). Using RMSE as the primary criterion, the PLS-SEM predictions yielded lower RMSE than LM for all seven indicators (ΔRMSE <0 throughout). MAE comparisons were mixed: PLS-SEM produced lower MAE for three indicators and slightly higher MAE for the remaining indicators. Taken together, the PLSpredict results suggest that the estimated model provides meaningful out-of-sample predictive information for both the mediator (SEn) and the outcome (ET), complementing the model’s explanatory results (*R*^2^_SEn_ = 0.336; *R*^2^_ET_ = 0.593). Given the cross-sectional, self-report design, these structural relations should be interpreted as associations rather than causal effects.

## 5 Discussion

### 5.1 Summary of main findings

This study examined a psychological pathway from academic self-efficacy to engineering thinking in DBL-implemented undergraduate engineering courses. Self-efficacy positively predicted student engagement, and engagement was strongly associated with engineering thinking. Self-efficacy also retained a smaller but significant direct association with engineering thinking. Mediation results indicate complementary partial mediation (Zhao et al., 2010); descriptively, the indirect effect accounted for about 62% of the total effect (VAF). VAF is reported as a descriptive proportion rather than as a decision rule. The model explained meaningful variance in both engagement (*R*^2^ = 0.336) and engineering thinking (*R*^2^ = 0.593), and PLSpredict results suggested out-of-sample predictive relevance for the mediator and outcome.

### 5.2 Theoretical implications

The findings extend social cognitive and engagement perspectives on how self-efficacy, student engagement, and design-based learning contribute to the development of engineering thinking in undergraduate engineering education. First, the strong self-efficacy → engagement path is consistent with Social Cognitive Theory, which posits that self-efficacy regulates the amount of effort and perseverance students invest in learning tasks (Bandura, 1997; Chemers et al., 2001; Eccles and Wigfield, 2002). The results echo prior work showing that academic self-efficacy predicts different facets of engagement in higher education, including in Asian and other non-Western contexts (Luo et al., 2023; Wang and Zhang, 2024). By modeling engagement as a higher-order construct with behavioral, emotional, and cognitive dimensions, the present study reinforces the view that self-efficacy energizes not only visible participation, but also interest, enjoyment, and strategic thinking in design-focused courses (English and Kitsantas, 2013; Linnenbrink and Pintrich, 2003).

Second, the engagement → engineering thinking path supports arguments that design-oriented and project-based pedagogies foster complex cognitive outcomes largely through their impact on students’ active involvement in learning. Research on design-based and problem-based learning has shown that cognitive engagement can mediate the link between design-based activities and learning outcomes, and that systematic forms of engagement are particularly beneficial (English and Kitsantas, 2013; Fredricks et al., 2004; Wei et al., 2023). The present findings extend this line of work by operationalizing engineering thinking as an integrated higher-order construct—encompassing analytical, creative, systemic, and reflective dimensions—and demonstrating that engagement is a strong proximal predictor of this composite outcome in an applied university setting. Although engagement was modeled as a higher-order construct in this study, the findings are consistent with a more fine-grained mechanism in which self-efficacy supports students’ willingness to invest effort in design tasks (behavioral engagement), to experience interest and value in grappling with ill-structured problems (emotional engagement), and to apply deep strategies such as planning, monitoring, and reflecting on their work (cognitive engagement). These strands of engagement, in turn, provide the experiential basis for the analytical, creative, systemic, and reflective facets of engineering thinking. Future work could unpack this mechanism by examining which strands of engagement are most strongly linked to particular dimensions of engineering thinking.

Third, the partial mediation pattern aligns with broader evidence that engagement often mediates the relationship between self-efficacy and academic performance or perceived learning (Luo et al., 2023; Wang and Zhang, 2024). Focusing on engineering thinking within a design-based context helps to sharpen this mechanism. In the structural model, self-efficacy supports engineering thinking in two ways: directly, likely through its influence on goal setting, willingness to tackle complex design problems, and persistence under ambiguity; and indirectly, by encouraging richer engagement in iterative design tasks (Benlahcene et al., 2024; Luo et al., 2023). This twofold pattern suggests that engagement should be regarded not only as an outcome of instructional innovations, but also as a psychological process variable that connects students’ self-beliefs with domain-specific higher-order capabilities.

Methodologically, the use of a higher-order, multidimensional modeling approach adds to research on engineering thinking and engagement. Representing self-efficacy, engagement, and engineering thinking as higher-order constructs with multiple first-order dimensions allows the model to capture the theoretical complexity of these constructs while keeping the structural part relatively parsimonious. This specification accords with recent recommendations for modeling complex constructs in PLS-SEM and for assessing both explanatory power and predictive performance through procedures such as PLSpredict (Hair et al., 2022; Ringle et al., 2012; Shmueli et al., 2019).

### 5.3 Practical implications for design-based engineering education

The results suggest that interventions aimed at strengthening engineering thinking in DBL-implemented courses may be more effective when they support both students’ efficacy beliefs and their sustained engagement in iterative design work.

At the course-design level, instructors can increase mastery experiences by structuring DBL cycles into short, assessable iterations. A practical approach is to specify (a) clear design requirements and constraints at the outset, (b) intermediate deliverables aligned with each DBL phase (problem framing notes, concept sketches, prototype evidence, reflection logs), and (c) revision opportunities that make improvement visible. Brief “success criteria” checklists and exemplars can help students calibrate what counts as an acceptable solution at each iteration, which can reduce uncertainty and support efficacy.

In large classes, a scalable feedback strategy is to combine peer assessment with light-touch instructor moderation. Course teams can assign stable small groups, provide brief rubric training with one or two anchor examples, and require short justification comments for each rating to discourage superficial scoring. Reliability can be improved by using multiple peer raters per product and by applying instructor audits to a small random subset of submissions, with targeted re-scoring when ratings diverge substantially. This approach increases feedback volume without overloading instructors and can sustain behavioral and cognitive engagement across iterations.

To align engagement with specific facets of engineering thinking, instructors can embed structured prompts into DBL artifacts. For analytical thinking, require problem decomposition and requirement tracing; for creative thinking, require divergent–convergent ideation steps and rationale for concept selection; for systematic thinking, require a subsystem interaction map or trade-off table; and for reflective thinking, require post-iteration reflection on what failed, what evidence supports revisions, and what would be tested next. These routines create repeated opportunities for students to act on self-efficacy through engagement in design practices that are closely tied to engineering thinking outcomes.

### 5.4 China-specific contextual mechanisms

The findings should be interpreted against features of Chinese undergraduate engineering education that can shape how self-efficacy is translated into engagement during DBL-implemented coursework. First, many students enter university with learning habits formed in examination-oriented schooling, where correctness and efficiency are rewarded and uncertainty is penalized. DBL tasks, by contrast, require sustained work under ambiguity, iterative improvement, and tolerance of temporary failure. In such contexts, self-efficacy may become especially consequential because it supports persistence when goals and solution pathways are not fully specified.

Second, DBL implementation in application-oriented universities often occurs in large classes, which can constrain feedback density and limit opportunities for individualized coaching. When instructor feedback is infrequent, students must rely more heavily on peer interaction, group coordination, and self-regulation to maintain behavioral participation and cognitive investment. This can amplify the role of engagement as the day-to-day mechanism through which self-efficacy is expressed in learning activity.

Third, engineering programs in China have been encouraged to strengthen practice-oriented competencies under “New Engineering” initiatives (Zhuang and Xu, 2018). However, the translation of policy intent into classroom routines can be moderated by assessment systems that still prioritize high-stakes summative grading, uneven laboratory or maker-space resources, and coordination costs across courses. These constraints can shape both students’ perceived efficacy and the extent to which DBL cycles (problem exploration, ideation, prototyping, reflection) are implemented with sufficient time, scaffolding, and opportunities for revision.

### 5.5 Limitations and future research

Several limitations should be acknowledged. The cross-sectional design limits the strength of causal inferences. Although the hypothesized direction from self-efficacy to engagement and from engagement to engineering thinking is theoretically grounded, reciprocal relationships are also plausible (Linnenbrink and Pintrich, 2003). Longitudinal or experimental studies could track how self-efficacy, engagement, and engineering thinking co-develop over time in design-based courses and examine whether changes in self-efficacy precede subsequent changes in engagement and thinking, or vice versa.

All variables were measured via self-report, which raises concerns about common method variance and the accuracy of self-assessments of engineering thinking. The study addressed this issue through established scale development procedures and collinearity diagnostics, yet future work would benefit from incorporating performance-based indicators of engineering thinking (e.g., expert ratings of design products, analysis of design reports, or standardized assessments) and behavioral traces of engagement (Dym et al., 2005; Prince and Felder, 2006).

DBL was treated as a defining feature of the instructional context rather than a measured construct. While eligibility screening ensured verified exposure to core DBL phases, the study did not quantify implementation fidelity (e.g., intensity, scaffolding, feedback quality, and iteration opportunities) across courses and instructors. Accordingly, the reported coefficients should be interpreted as average associations across DBL-implemented courses, and they may differ under stronger or weaker DBL enactment.

The sample was drawn from a single teaching-focused university in eastern China, which also constrains generalizability to other institutional types, countries, disciplines, or technology-mediated delivery modes (e.g., online or hybrid learning) where engagement processes may differ (Maricuțoiu and Sulea, 2019; Nkomo et al., 2021; Stan et al., 2022). Future studies could validate the model across multiple institutions and engineering majors, and extend the framework by incorporating additional constructs such as engineering identity, interest, goal orientations, or perceptions of the learning environment (Luo et al., 2023; Wang and Zhang, 2024). Future studies could extend the present model by incorporating behavioral indicators of engagement (e.g., learning analytics traces) and performance-based assessments of engineering thinking to validate whether the observed associations hold beyond self-report data.

## 6 Conclusion

This study tested a self-efficacy → student engagement → engineering thinking mechanism among Chinese engineering undergraduates enrolled in DBL-implemented courses using a higher-order PLSc-based PLS-SEM mediation model. Self-efficacy positively predicted student engagement and engineering thinking, and the mediation results indicate complementary partial mediation, with self-efficacy linked to engineering thinking both directly and indirectly through engagement. The model demonstrated meaningful explanatory power (*R*^2^_SEn_ = 0.336; *R*^2^_ET_ = 0.593) and positive out-of-sample predictive relevance for both the mediator and outcome (*Q*^2^_predict_ ≈ 0.28). These findings highlight student engagement as a key psychological mechanism through which efficacy beliefs are linked to higher-order engineering thinking in design-oriented course contexts. Conclusions are tempered by the cross-sectional, self-report design and the single-institution sample; future studies should use longitudinal or experimental designs, incorporate performance-based indicators of engineering thinking and behavioral traces of engagement, and validate the model across institutions and engineering disciplines.

## Data Availability

The raw data supporting the conclusions of this article will be made available by the authors, without undue reservation.

## References

[ref1] Adams A.-M. Wilson H. Money J. Palmer-Conn S. Fearn J. (2020). Student engagement with feedback and attainment: the role of academic self-efficacy. Assess. Eval. High. Educ. 45, 317–329. doi: 10.1080/02602938.2019.1640184

[ref2] Azila-Gbettor E. M. Mensah C. Abiemo M. K. Bokor M. (2021). Predicting student engagement from self-efficacy and autonomous motivation: a cross-sectional study. Cogent Educ. 8:1942638. doi: 10.1080/2331186X.2021.1942638

[ref3] Bandura A. (1997). Self-Efficacy: The Exercise of Control. New York, NY: W. H. Freeman.

[ref4] Benlahcene A. Mohamed Abdelrahman R. Ahmed M. Aboudahr S. M. F. M. (2024). A pathway to engagement: the mediating role of self-efficacy between interpersonal relationships and academic engagement. Cogent Psychol. 11:2330239. doi: 10.1080/23311908.2024.2330239

[ref5] Chemers M. M. Hu L. Garcia B. F. (2001). Academic self-efficacy and first-year college student performance and adjustment. J. Educ. Psychol. 93, 55–64. doi: 10.1037/0022-0663.93.1.55

[ref6] Dym C. L. Agogino A. M. Eris O. Frey D. D. Leifer L. J. (2005). Engineering design thinking, teaching, and learning. J. Eng. Educ. 94, 103–120. doi: 10.1002/j.2168-9830.2005.tb00832.x

[ref7] Eccles J. S. Wigfield A. (2002). Motivational beliefs, values, and goals. Annu. Rev. Psychol. 53, 109–132. doi: 10.1146/annurev.psych.53.100901.135153, 11752481

[ref8] English M. C. Kitsantas A. (2013). Supporting student self-regulated learning in problem- and project-based learning. Interdiscip. J. Probl.-Based Learn. 7, 128–150. doi: 10.7771/1541-5015.1339

[ref9] Fredricks J. A. Blumenfeld P. C. Paris A. H. (2004). School engagement: potential of the concept, state of the evidence. Rev. Educ. Res. 74, 59–109. doi: 10.3102/00346543074001059

[ref10] Fredricks J. A. Filsecker M. Lawson M. A. (2016). Student engagement, context, and adjustment: addressing definitional, measurement, and methodological issues. Learn. Instr. 43, 1–4. doi: 10.1016/j.learninstruc.2016.02.002

[ref11] Gómez Puente S. M. van Eijck M. Jochems W. (2015). Professional development for design-based learning in engineering education: a case study. Eur. J. Eng. Educ. 40, 14–31. doi: 10.1080/03043797.2014.903228

[ref12] Hair J. F. Hult G. T. M. Ringle C. M. Sarstedt M. (2022). A Primer on Partial Least Squares Structural Equation Modeling (PLS-SEM). Thousand Oaks, CA: SAGE.

[ref13] Hmelo-Silver C. E. (2004). Problem-based learning: What and how do students learn? Educ. Psychol. Rev. 16, 235–266. doi: 10.1023/B:EDPR.0000034022.16470.f3

[ref14] Honicke T. Broadbent J. (2016). The influence of academic self-efficacy on academic performance: a systematic review. Educ. Res. Rev. 17, 63–84. doi: 10.1016/j.edurev.2015.11.002

[ref15] Kock N. (2015). Common method bias in PLS-SEM: a full collinearity assessment approach. Int. J. E-Collab. 11, 1–10. doi: 10.4018/ijec.2015100101

[ref16] Lam S. Wong B. P. H. Yang H. Liu Y. (2012). “Understanding student engagement with a contextual model,” in Handbook of Research on Student Engagement, eds. Christenson S. L. Reschly A. L. Wylie C. (Berlin: Springer).

[ref17] Linnenbrink E. A. Pintrich P. R. (2003). The role of self-efficacy beliefs in student engagement and learning in the classroom. Read. Writ. Q. 19, 119–137. doi: 10.1080/10573560308223

[ref18] Luo Q. Chen L. Yu D. Zhang K. (2023). The mediating role of learning engagement between self-efficacy and academic achievement among Chinese college students. Psychol. Res. Behav. Manag. 16, 1533–1543. doi: 10.2147/PRBM.S401145, 37143904 PMC10153452

[ref19] Mamaril N. A. Usher E. L. Li C. R. Economy D. R. Kennedy M. S. (2016). Measuring undergraduate students’ engineering self-efficacy: a validation study. J. Eng. Educ. 105, 366–395. doi: 10.1002/jee.20121

[ref20] Maricuțoiu L. P. Sulea C. (2019). Evolution of self-efficacy, student engagement and student burnout during a semester. A multilevel structural equation modeling approach. Learn. Individ. Differ. 76:101785. doi: 10.1016/j.lindif.2019.101785

[ref21] Nkomo L. M. Daniel B. K. Butson R. J. (2021). Synthesis of student engagement with digital technologies: a systematic review of the literature. Int. J. Educ. Technol. High. Educ. 18:34. doi: 10.1186/s41239-021-00270-1, 34778529 PMC8241468

[ref22] Prince M. J. Felder R. M. (2006). Inductive teaching and learning methods: definitions, comparisons, and research bases. J. Eng. Educ. 95, 123–138. doi: 10.1002/j.2168-9830.2006.tb00884.x

[ref23] Reeve J. Tseng C.-M. (2011). Agency as a fourth aspect of students’ engagement during learning activities. Contemp. Educ. Psychol. 36, 257–267. doi: 10.1016/j.cedpsych.2011.05.002

[ref24] Richardson M. Abraham C. Bond R. (2012). Psychological correlates of university students’ academic performance: a systematic review and meta-analysis. Psychol. Bull. 138, 353–387. doi: 10.1037/a0026838, 22352812

[ref25] Ringle C. M. Sarstedt M. Straub D. W. (2012). Editor’s comments: a critical look at the use of PLS-SEM in MIS quarterly. MIS Q. 36, iii–xiv. doi: 10.2307/41410402

[ref26] Schaufeli W. B. Martínez I. M. Pinto A. M. Salanova M. Bakker A. B. (2002). Burnout and engagement in university students: a cross-national study. J. Cross-Cult. Psychol. 33, 464–481. doi: 10.1177/0022022102033005003

[ref27] Shmueli G. Sarstedt M. Hair J. F. Cheah J.-H. Ting H. Vaithilingam S. . (2019). Predictive model assessment in PLS-SEM: guidelines for using PLSpredict. Eur. J. Mark. 53, 2322–2347. doi: 10.1108/EJM-02-2019-0189

[ref28] Stan M. M. Topală I. R. Necşoi D. V. Cazan A.-M. (2022). Predictors of learning engagement in the context of online learning during the COVID-19 pandemic. Front. Psychol. 13:867122. doi: 10.3389/fpsyg.2022.867122, 35572259 PMC9100394

[ref29] Wang Y. Zhang W. (2024). The relationship between college students’ learning engagement and academic self-efficacy: a moderated mediation model. Front. Psychol. 15:1425172. doi: 10.3389/fpsyg.2024.1425172, 39291178 PMC11407112

[ref30] Wei L. Zhang W. Lin C. (2023). The study of the effectiveness of design-based engineering learning: the mediating role of cognitive engagement and the moderating role of modes of engagement. Front. Psychol. 14:1151610. doi: 10.3389/fpsyg.2023.1151610, 37303900 PMC10250629

[ref31] Zhao X. Lynch J. G. Chen Q. (2010). Reconsidering Baron and Kenny: myths and truths about mediation analysis. J. Consum. Res. 37, 197–206. doi: 10.1086/651257

[ref32] Zhuang T. Xu X. (2018). “New engineering education” in Chinese higher education: prospects and challenges. Tun. J. High. Educ. 6, 69–109. doi: 10.18543/tjhe-6(1)-2018pp69-109

