# Supplementary material for: From self-efficacy to engineering thinking: the mediating role of student engagement among undergraduate engineering students in China
Source: Front Psychol. 2026 Mar 20;17:1761444. doi: 10.3389/fpsyg.2026.1761444 (PMC13047130; doi:10.3389/fpsyg.2026.1761444)
Supplement: Supplementary file 1 [file Table_1.docx]

Supplementary Table S1
Full wording of the 36 survey items, dimension labels, and source/adaptation notes for Self-Efficacy (SE), Student Engagement (SEn), and Engineering Thinking (ET).

| **Construct** | **Dimension** | **Item code** | **Final item wording** | Source/adaptation notes |
| --- | --- | --- | --- | --- |
| Self-Efficacy (SE) | Task-Specific Self-Efficacy | SE_TS_1 | I am confident that I can successfully complete the learning tasks required in engineering learning activities. | Adapted/assembled from academic and engineering self-efficacy literature (e.g., Bandura, 1997; Chemers et al., 2001; Mamaril et al., 2016); refined for DBL-implemented engineering course contexts. |
| Self-Efficacy (SE) | Task-Specific Self-Efficacy | SE_TS_2 | I am confident that I can acquire the skills needed to meet learning requirements in engineering learning activities. |  |
| Self-Efficacy (SE) | Task-Specific Self-Efficacy | SE_TS_3 | When tasks are difficult, I am confident that I can persist and complete them. |  |
| Self-Efficacy (SE) | Context-Specific Self-Efficacy | SE_CS_1 | I am confident that I can perform effectively across different learning situations used in engineering learning activities. |  |
| Self-Efficacy (SE) | Context-Specific Self-Efficacy | SE_CS_2 | I am confident that I can respond effectively to challenges that arise in different classroom situations. |  |
| Self-Efficacy (SE) | Context-Specific Self-Efficacy | SE_CS_3 | I am confident that I can remain focused and effective across different instructional settings. |  |
| Self-Efficacy (SE) | Affective Self-Efficacy | SE_AF_1 | I am confident that I can maintain emotional stability when facing learning pressure. |  |
| Self-Efficacy (SE) | Affective Self-Efficacy | SE_AF_2 | I am confident that I can regulate anxiety so that it does not hinder my learning. |  |
| Self-Efficacy (SE) | Affective Self-Efficacy | SE_AF_3 | After setbacks, I am confident that I can regain motivation to study. |  |
| Self-Efficacy (SE) | Time Management Self-Efficacy | SE_TM_1 | I am confident that I can allocate my study time effectively to complete tasks on schedule. |  |
| Self-Efficacy (SE) | Time Management Self-Efficacy | SE_TM_2 | I am confident that I can make a study plan and follow it consistently. |  |
| Self-Efficacy (SE) | Time Management Self-Efficacy | SE_TM_3 | I am confident that I can stay on task and meet deadlines even when time is limited. |  |
| Self-Efficacy (SE) | Self-Regulation Self-Efficacy | SE_SR_1 | I am confident that I can monitor my learning progress and make necessary adjustments. |  |
| Self-Efficacy (SE) | Self-Regulation Self-Efficacy | SE_SR_2 | I am confident that I can adapt my learning strategies based on feedback and results. |  |
| Self-Efficacy (SE) | Self-Regulation Self-Efficacy | SE_SR_3 | I am confident that I can overcome obstacles in learning through self-directed effort. |  |
| Student Engagement (SEn) | Behavioral Engagement | SEn_BE_1 | I stay focused on learning tasks during engineering learning activities and avoid distractions. | Adapted/assembled from multidimensional student engagement frameworks and higher education engagement research (e.g., Fredricks et al., 2004, 2016; Reeve & Tseng, 2011); refined for DBL-implemented engineering course contexts. |
| Student Engagement (SEn) | Behavioral Engagement | SEn_BE_2 | I complete assigned learning tasks on time in engineering learning activities. |  |
| Student Engagement (SEn) | Behavioral Engagement | SEn_BE_3 | I actively participate in engineering learning activities, such as asking or answering questions and engaging in group work. |  |
| Student Engagement (SEn) | Emotional Engagement | SEn_EM_1 | I feel enthusiastic and interested during engineering learning activities. |  |
| Student Engagement (SEn) | Emotional Engagement | SEn_EM_2 | The content of engineering learning activities makes me feel interested and personally involved. |  |
| Student Engagement (SEn) | Emotional Engagement | SEn_EM_3 | I enjoy participating in engineering learning activities. |  |
| Student Engagement (SEn) | Cognitive Engagement | SEn_CO_1 | I try to understand the learning content in depth and connect it with what I already know. |  |
| Student Engagement (SEn) | Cognitive Engagement | SEn_CO_2 | I think carefully about the questions raised and form my own explanations. |  |
| Student Engagement (SEn) | Cognitive Engagement | SEn_CO_3 | I invest additional mental effort to understand the topics more thoroughly, especially when they are challenging. |  |
| Engineering Thinking (ET) | Analytical Thinking | ET_AN_1 | When solving an engineering problem, I can identify its core technical issue. | Adapted/assembled from engineering design thinking and related STEM education literature (e.g., Dym et al., 2005; Prince & Felder, 2006; Wei et al., 2023); refined for self-reported engineering thinking in DBL-implemented engineering course contexts. |
| Engineering Thinking (ET) | Analytical Thinking | ET_AN_2 | When solving an engineering problem, I can break a complex problem into manageable parts and identify its key elements. |  |
| Engineering Thinking (ET) | Analytical Thinking | ET_AN_3 | When solving an engineering problem, I can use logical reasoning to evaluate alternatives and justify decisions. |  |
| Engineering Thinking (ET) | Creative Thinking | ET_CR_1 | I can generate original and feasible ideas for engineering design solutions. |  |
| Engineering Thinking (ET) | Creative Thinking | ET_CR_2 | Before deciding on a solution, I can generate multiple possible approaches to an engineering problem. |  |
| Engineering Thinking (ET) | Creative Thinking | ET_CR_3 | I can combine ideas in new ways to develop better engineering solutions. |  |
| Engineering Thinking (ET) | Systematic Thinking | ET_SY_1 | When solving an engineering problem, I consider relationships among components and subsystems. |  |
| Engineering Thinking (ET) | Systematic Thinking | ET_SY_2 | I can integrate different types of information to form a comprehensive understanding of an engineering problem. |  |
| Engineering Thinking (ET) | Systematic Thinking | ET_SY_3 | I can analyze an engineering problem from an overall system perspective rather than focusing only on isolated parts. |  |
| Engineering Thinking (ET) | Reflective Thinking | ET_RE_1 | After completing an engineering task, I reflect on both the process and the decisions I made. |  |
| Engineering Thinking (ET) | Reflective Thinking | ET_RE_2 | I can analyze reasons for success or failure in order to improve my future engineering work. |  |
| Engineering Thinking (ET) | Reflective Thinking | ET_RE_3 | I can learn from previous engineering experiences and apply that learning to future practice. |  |

***Note.*** All items were rated on a five-point Likert scale ranging from 1 (strongly disagree) to 5 (strongly agree).
